# Research on the Wear Suppression of Diamond Grain Enabled by Hexagonal Boron Nitride in Grinding Cast Steel

**DOI:** 10.3390/molecules29245925

**Published:** 2024-12-16

**Authors:** Hongrui Zhao, Qun Sun, Chong Wang, Xiuhua Yuan, Xia Li

**Affiliations:** 1 School of Mechanical and Automotive Engineering, Liaocheng University, Liaocheng 252000, China; 2320230122@stu.lcu.edu.cn (H.Z.); sunqun@lcu.edu.cn (Q.S.); wangchong@lcu.edu.cn (C.W.); 2 School of Chemistry and Chemical Engineering, Liaocheng University, Liaocheng 252000, China

**Keywords:** diamond grain, grinding, hexagonal boron nitride, wear suppression, molecular dynamics

## Abstract

Diamond grinding wheels have been widely used to remove the residual features of cast parts, such as parting lines and pouring risers. However, diamond grains are prone to chemical wear as a result of their strong interaction with ferrous metals. To mitigate this wear, this study proposes the use of a novel water-based hexagonal boron nitride (hBN) as a minimum quantity lubrication (MQL) during the grinding of cast steel and conducted the grinding experiment and molecular dynamics simulation. The experiment demonstrated that compared to dry grinding, the water-based hBN nanofluid can effectively reduce the maximum temperature of a workpiece at contact zone from 408 K to 335 K and change the serious abrasion wear of diamond grain to slightly micro-broken. The molecular dynamics simulation indicates that the flake of hBN can weaken the catalytic effect of iron on the diamond, prevent the diffusion of carbon atom to cast steel, and suppress the graphitization of diamond grain. Additionally, the flake of hBN improves the contact state between the diamond grain and cast steel and reduces the cutting heat and friction coefficient from about 0.5 to 0.25. Thus, the water-based hBN nanofluid as a new MQL was proven to be suitable for the wear inhibition of diamond grain when grinding cast steel.

## 1. Introduction

The casting process [1] shapes liquid metal into functional parts with complex structures, with applications in the field of automobiles, aircraft and ships. Affected by the casting process, residual features such as pouring risers and the parting lines of casting blanks will appear [2]. To obtain the final casting parts, the grinding of these residual features [3,4] is the indispensable primary process in producing casting parts. Currently, robots [5] equipped with diamond grain have been developed for the grinding of casting parts. Although the diamond wheel has been successfully applied in dry grinding cast iron with a long service life [6,7,8,9], there is a serious problem of mechanic-chemical wear in grinding cast steel. Consequently, the wear resistance of the diamond grains are the main factors on the service life of the grinding wheel.

When cutting ferrous metals, the diamond tool experienced a series of chemical reactions, leading to excessive wear [10]. It was generally believed that in high-temperature and high-pressure environment, the atoms of diamonds can easily diffuse into the interstitial locations of the iron lattice and convert into graphite under the catalysis of iron elements [11,12]. The MQL-assisted grinding under fluid spray can both effectively reduce tool wear and improve the machined surface quality [13,14]. Compared with flood grinding, the MQL can effectively reduce harmful residues and lower production cost [15]. Moreover, various nanoparticles, such as carbon nanotube [16], graphene [17], and fullerene [18], were dispersed in cutting fluid to prepare high-performance nanofluids. Although the above nanomaterials can reduce friction, the atom diffusion wear of diamonds still exists [19]. Therefore, it was urgent to apply new nanomaterials to the cutting of nanofluids and explore the wear inhibition of diamonds during mist-spray lubrication grinding.

The hBN, as a two-dimensional (2D) ceramic nanomaterial, exhibited excellent oxidation resistance, low sliding friction, and good thermal stability. The hBN was commonly applied in metallic composites via sintering, which exhibited a self-lubricating ability [20]. In order to improve the tribological property of lubricants, the hBN was dispersed to the organic solvents [21,22]. The conventional nanofluids were mostly oil-based [23,24], which not only showed low thermal conductivity and poor permeability, but also added to the environmental burden. Recently, the environmentally friendly water-based hBN nanofluid [25] was applied in the milling process of tungsten carbide cutters, and it was able to improve the milling quality of Ti-6Al-4V workpiece; this nanofluid was also applied to grinding alumina ceramic, and it was able to reduce friction and residual stresses [26]. To date, there is no study on using water-based hBN nanofluid to suppress diamond wear, especially under the grinding environment of ferrous metal. Meanwhile, there is a lack of mechanism exploration in terms of hBN wear suppression from an atomic perspective. Therefore, in order to suppress the wear of diamond grain and extend its service life, this paper proposed the use of a novel water-based hexagonal boron nitride (hBN) as a minimum quantity lubrication (MQL) in grinding cast steel and conducted the grinding experiment and molecular dynamics simulations. The suppression performance of hBN on diamond wear was evaluated in terms of wear morphology, dynamic properties, and atomic structure phase transition, and its suppression mechanism on diamond wear from an atomic perspective was also explained in detail.

## 2. Materials and Methods

### 2.1. Experiment Setup

**Figure 1:**
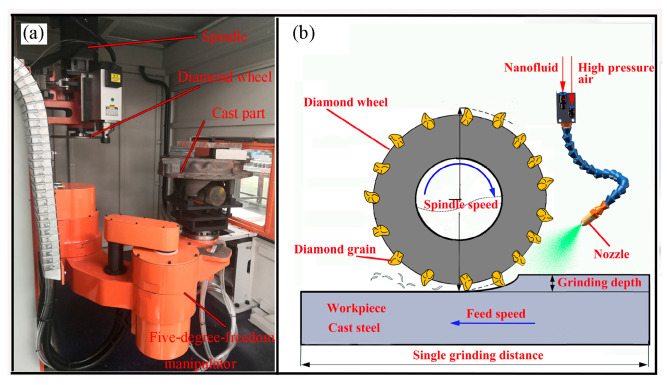
Robotic grinding platform (**a**) and grinding experiment (**b**).

The grinding experiment was conducted using a self-made robotic automatic platform (Figure 1a) which was mainly composed of a 5-DOF robot arm, robot controller, servo motor, spindle, diamond wheel, MQL system, etc. The revolute joint of the robot arm was equipped with the harmonic reducer (T-355, Tokyo, Japan) and was driven by a servo motor (MGMF132L1H6M, Tokyo, Japan) operating in the current control mode. The robot arm was controlled by an industrial controller (LNC Technology Co., Ltd., R8800D3, Taizhong, China) with a handling capacity of 20 kg and 1.0 m reach. Before the grinding process, the operator applied teach-playback operating mode to teach the contours of the cast part to the robot. Subsequently, the robotic platform automatically grabbed the cast part and ground it along a contour on the diamond wheel. Therefore, it has high flexibility and is suitable for grinding cast parts with various morphological characteristics.

**Figure 2:**
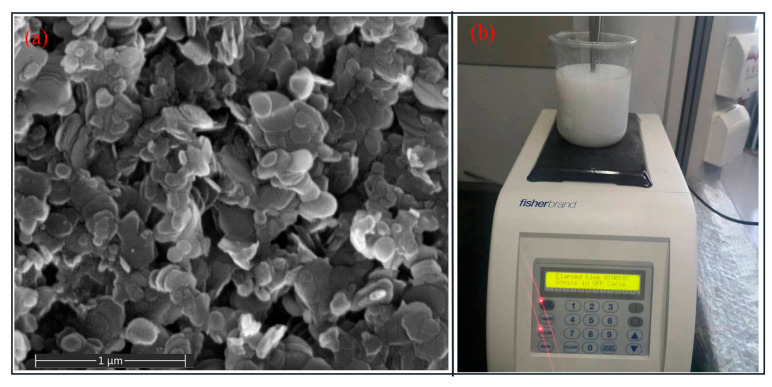
Morphology of hBN (**a**) and ultrasonic dispersion (**b**).

The MQL system was composed of an air compressor, pressure regulator, flow meter, and application nozzle. As shown in Figure 1b, the MQL nanofluid was applied through the nozzle positioned 40 mm away from the cut zone. As shown in Figure 2a, the hBN exhibited a typical two-dimensional layer sheet-like structure with a diameter in the range of several hundred nanometers. In this study, the preparation process of the hBN nanofluid was as follows: firstly, the hBN and sodium dodecyl sulfate with mass fractions of 0.2%, 1%, respectively, were thoroughly mixed in the deionized water by magnetic stirring for 1 h; subsequently, the mixture was subjected to ultrasonic vibration for 30 min (40 kHz, 60 W, Figure 2b) to avoid agglomeration and deposition. Although the hBN tends to aggregate, the hBN nanofluid was still stable after resting for 24 h.

**Figure A1:**
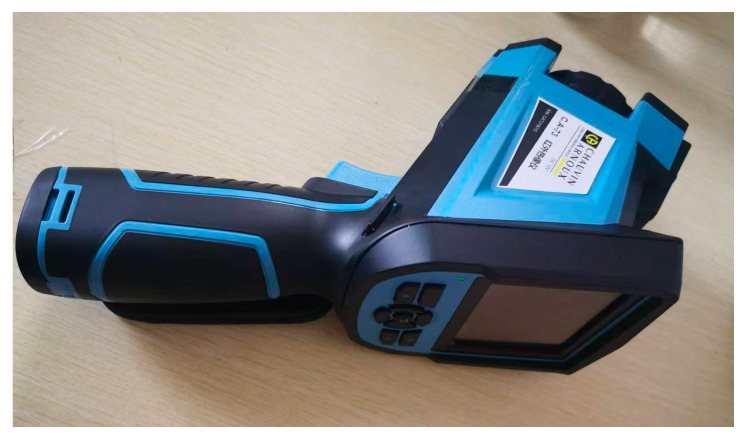
The infrared camera.

The grinding experiments were conducted to evaluate the effect of the as-prepared nanofluid on the diamond grain. As shown in Figure 1b, the plane of the workpiece was ground by the outer circle of the diamond wheel, which was flat surface grinding. The workpiece was the J03000 cast steel with dimensions of 300 mm × 200 mm × 40 mm. The grinding wheel was a braze diamond wheel with a 35-mesh diamond grain, 65 Mn steel substrate, and 250 mm diameter. The mono-layer diamond grain was brazed to the 65 Mn steel substrate by Ni-Cr alloy. The grinding parameters were as follows: the grinding speed, feed rates, and grinding depth were 30 m/s, 200 mm/min, and 20 μm, respectively. The single grinding distance, which was the length of the diamond wheel moving in the feed direction at once, was 300 mm of the length of the workpiece. During the water-based hBN nanofluid spray, the compressed air and nanofluid were mixed in a nozzle, and atomized particles were sprayed into the grinding zone, which is shown in Figure 1b. The nozzle was 40 mm away from the grinding plane, and its axis was a 30° angle with the grinding plane so that the nanofluid was uniformly injected into the grinding zone. The air pressure and flow rate of the nanofluid were 0.5 Mpa and 120 mL/h, respectively. After the total grinding length of 1 km, the wear morphology and the typical elements of the diamond grain were characterized by scanning electron microscopy (SEM). The SEM (Zeiss Merlin Compact, Carl Zeiss Company, Oberkochen, Germany) was equipped with a hot-filed emission electron gun with an acceleration voltage of 0.05 kV to 30 kV and a probe current of 5 pA to 20 nA. The SEM can analyze the element type and content from beryllium to americium. The grinding temperature was monitored using an infrared camera (Chauin Arnoux Group, C.A-73, Paris, France), which was shown in Figure A1. The infrared camera can detect infrared waves ranging from 7.5 μm to 14 μm, possessing 0.13° spatial resolution. It can detect the temperature from −20–650 °C with a thermal sensitivity of 0.06 °C@30 °C.

### 2.2. Simulation Setup

**Figure 3:**
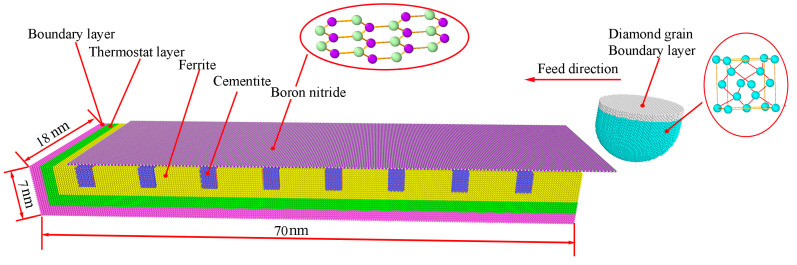
Molecular dynamics model.

The water-based hBN nanofliud was mainly composed of water molecules and the hBN. According previous simulation reports [27,28], water molecules can effectively absorb grinding heat and reduce grinding temperature, but they cannot change the contact state between the workpiece surface and diamond grain. On the other hand, the flake of hBN was an excellent lubricant and can reduce the friction force. So, this section mainly explores the wear inhibition effect of hBN on diamond grain. It is well known that the abrasive grain presents a large negative rake angle to the workpiece, and the negative rake angle of cutting tool can effectively influence the material removal or deformation mechanisms [29]. During the grinding process, only a very small fraction (18%) of the grains merely rub or plow into the work material and even a smaller fraction (1.8%) of that participate in actual shearing [30]. Moreover, unlike turning or milling, it was difficult to confirm the accurate value of the negative rake angle of the diamond grain. Based on the above consideration, this paper simplified diamond grains to the shapes of hemispheres and cylinders. The large-scale atomic/molecular massively parallel simulator (LAMMPS) [31] and the OVITO [32] were applied to simulate and visualize the grinding process, respectively. The grinding model was shown in Figure 3 with lattice directions of x [100], y [010], and z [001], respectively. The workpiece was comprised of two lattices: orthorhombic lattice of cementite and body-centered cubic lattice of iron, due to the fact that the cast steel was mainly composed of pearlite and ferrite, and the diamond grain was composed of carbon with diamond cubic lattice. The workpiece matrix was divided into three regions: a boundary layer, a constant temperature layer, and a newton layer. Similarly, the abrasive grain was divided into two regions: the newton layer and the boundary layer. The atoms at the boundary layer were fixed so that the diamond grain and workpiece did not deviate from the preset position. The atoms in the thermostatic layer were set to a temperature of 300 K to dissipate the heat generated by grinding. The atoms at the newton layer were set as deformable body to study the grinding process. When grinding ferrous metal, the main causes of wear of the diamond grain were atom diffuses and lattice transformations [10,11]. Restricted by computing power, the molecular dynamics simulation can only evaluate the grinding process from the atomic and molecular levels (nm) [33]; therefore, this paper scaled back the diamond grain to the nanometer level, which was shown in Table 1. This scaling to the nanoscale cannot affect the analysis of atomic diffusion, lattice transition, temperature, stress, and material removal. Before the grinding process, the initial workpiece was relaxed to accomplish the thermal equilibrium state and diminish excess energy of the whole system under the isobaric-isothermal ensemble (NVE) at a temperature of 300 K in 120 ps.

The system model was divided into three parts: workpiece, diamond grain, and lubricant, consisting of four elements, namely Fe, C, B, and N. This study employed the modified embedded atom method (Meam potential [34]) to model the interaction between Fe and C atoms in the workpiece and diamond grain, shown as follows:

(1) $$E=\sum_{i}\left[F_{i}\left(\bar{\rho_{i}}\right)+\frac{1}{2}\sum_{j\neq i}S_{ij}\emptyset_{ij}\left(R_{ij}\right)\right]$$

where *F_i_* is the energy needed to embed atom *i* into the background electron density, $\bar{\rho_{i}}$ and $\emptyset_{ij}$ are the pair interaction term between atoms separated by the vector $R_{ij}$. The Tersoff potential [35] was applied to describe the interaction between B and N atoms in same layer, shown as follows:

(2) $$E=\frac{1}{2}\sum_{i}\sum_{i\neq j}f_{c}\left(r_{ij}\right)\left[f_{R}\left(r_{ij}\right)+b_{ij}f_{A}\left(r_{ij}\right)\right]$$

where $f_{c}\left(r_{ij}\right)$ is a cutoff function, $f_{R}\left(r_{ij}\right)$ and $f_{A}\left(r_{ij}\right)$ are the repulsive and attractive components of the potential, respectively, and $b_{ij}$ is a monotonically decreasing function. The inter-layer potential [35] between B and N atoms in different layers is shown as follows:

(3) $$E\left(r_{ij},\;\rho_{i}\right)=\mathrm{Tap}\left(r_{ij}\right)e^{\alpha_{ij}\left(1-\frac{r_{ij}}{\beta_{ij}}\right)}\left[\varepsilon_{ij}+C_{ij}\left(e^{-{\left(\frac{\rho_{ij}}{\gamma_{ij}}\right)}^{2}}+e^{{\left(\frac{\rho_{ij}}{\gamma_{ij}}\right)}^{2}}\right)\right]$$

where $\varepsilon_{ij}$ and $C_{ij}$ are energy constants associated with the isotropic and anisotropic repulsion, respectively, $\beta_{ij}$ and $\gamma_{ij}$ set the corresponding interaction ranges, and $\alpha_{ij}$ sets the steepness of the isotropic term. The atomic interactions among diamond grain, workpiece and lubricate were modeled by a 12-6 Lenard Jones potential, shown as follows:

(4) $$E=4\;\varepsilon_{ij}\left[{\left(\frac{\sigma_{ij}}{\gamma }\right)}^{12}-{\left(\frac{\sigma_{ij}}{\gamma }\right)}^{6}\right]$$

where $\sigma_{ij}$ represents the regulation parameter when the interaction potential energy is equal to 0, $\varepsilon_{ij}$ is the depth of the potential well, and γ is the atomic spacing.

## 3. Result and Discussion

### 3.1. Analysis of Grinding Process

**Figure 4:**
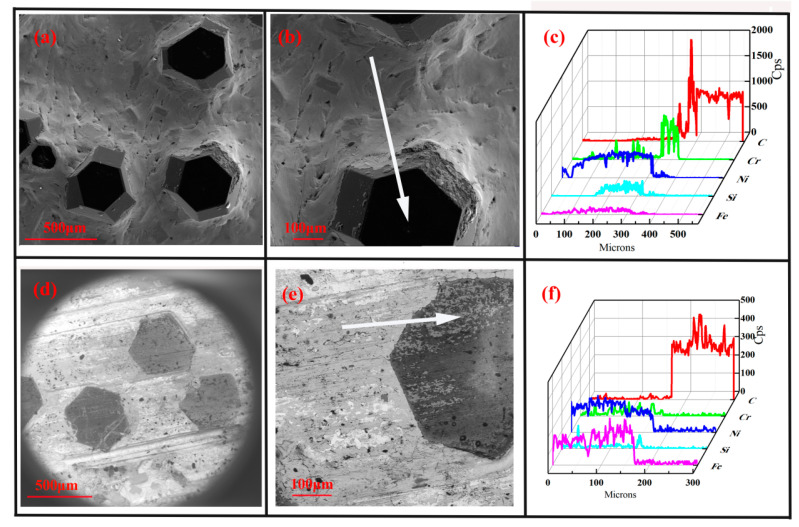
The morphology and element distribution (along white arrow) of diamond grain: before grinding (**a**–**c**), after dry grinding (**d**–**f**).

**Figure 5:**
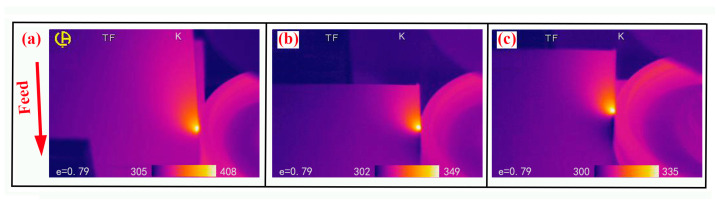
Temperature distribution: (**a**) dry grinding, (**b**) water-based spray, and water-based hBN spray (**c**).

The brazed diamond wheel was applied to the grinding cast steel experiment, and its morphology was shown in Figure 4. Before grinding, the diamond grain (Figure 4a,b) exhibited the cutting edge and surface integrity well, without cracks and holes. The diamond grain was well embedded in the active filler layer, which shows sufficient wetting ability on the diamond interface. As shown in Figure 4c, the active filler alloys mainly consisted of Ni and Cr elements. The Cr element diffused amplitude with a length of 40 μm toward the interface, and its counts were higher than that of the Ni element along the interface. After dry grinding for 1 km distance, the diamond grain (Figure 4d,e) experienced chemical wear and tear, which was similar to the previous report [36]. Its height was almost equal to that of the active filler layer, resulting in losing the grinding ability. As shown in Figure 4f, the worn grain mainly contained carbon elements, and its worn surface was adhered by a certain number of carbides (iron carbide, chromium carbide), which was consistent with the previous report [6]. As shown in Figure 5a, the maximum temperatures at grinding zones were 408 K and were significantly higher than that at matrix. It is worth noting that although the infrared thermal imager can only detect the temperature on the external surface of the workpiece, not inside of the diamond grain, it can display the changes in grinding temperature field under different working conditions. During the dry grinding, the higher temperature and pressure increased the energy of carbon atom at contact zone, resulting in the breakage of covalent bond. Especially under the catalysis of the vacancy electron on the d-orbitals of Fe atom, the atom of diamond at contact zone could easily diffuse into the workpiece, resulting in the rapid wear (adhesive wear, abrasion wear). Therefore, during the dry grinding cast steel, the diamond grain suffered from the serious mechanic-chemical wear, resulting in a shortened service life.

**Figure 6:**
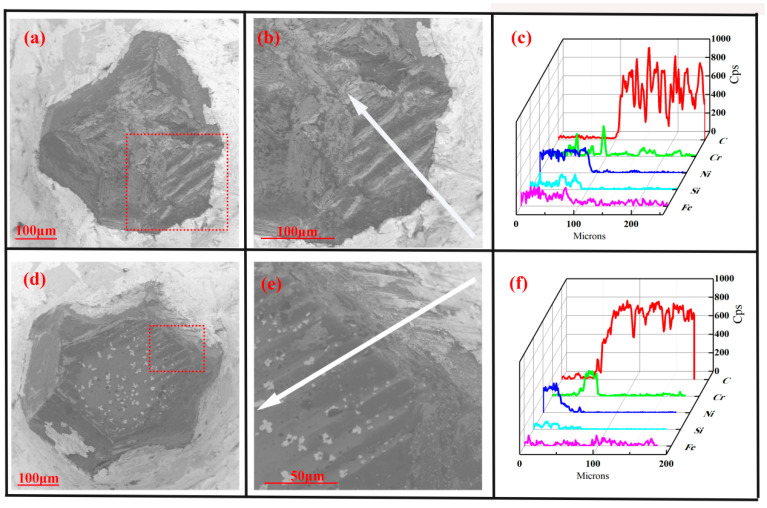
The morphology and element distribution (along white arrow) of diamond grain after water-based spray (**a**–**c**), and water-based hBN spray (**d**–**f**).

During the water-based spray, which did not contain the hBN nanoparticle, the grinding temperature filed was shown in Figure 5b, and the maximum temperatures at grinding zones were 349 K. Compared to dry grinding, the grinding heat was well dissipated. This can be explained by the fact that water can be better adsorbed to the surface of the workpiece due to it being a polar molecule. It can absorb the grinding heat, and take grinding heat away with high speed rotation. After wet grinding, the morphology of the diamond grains were shown in Figure 6a,b. The diamond grain showed the broken, fracture, and abrasion wear phenomenon on the surface, which was similar to previous reports [6,7]. It can be considered that grain breaking was the main phenomena, rather than grain detachment from the active filler layer. The fracture surface of the diamond grain was adhered by a small amount of metallic element (iron, chromium, Figure 6c), and the similar phenomenon has been observed in a previous report [8]. This can be explained by the fact that the periodical intense squeezing and friction generated the alternating mechanical and thermal stress, leading to the fatigue cracks and local damage of diamond grain.

During the water-based hBN spray, the grinding temperature filed was shown in Figure 5c, and the maximum temperature at grinding zones was reduced to 335 K, which was slightly lower to that of the water-based spray. This can be explained by the fact that the grinding heat mainly came from the elastic and plastic deformation of the workpiece [37], which was transferred out by water molecule, and the small portion of grinding heat came from friction between the diamond grain and workpiece. After grinding, the morphology of diamond grain was shown in Figure 6d,e. The diamond grain showed slightly broken and abrasion wear phenomenon on the surface. As shown in Figure 6f, a small amount of metal elements (iron, chromium) were attached to the fracture surface of diamond grain, which may originate from the debris adhering to the diamond grain during the grinding process. Compared to dry grinding, the low temperature can decrease the energy of the carbon atom, resulting in lower diffusion wear. Especially under the flake of hBN, the direct contact between diamond grain and workpiece was changed to an indirect contact (soft contact), and the catalysis of Fe atom on the diamond atom was suppressed. So, the water-based hBN spray can effectively reduce the wear of diamond grain and extend the service life of diamond wheel.

### 3.2. Simulation Analysis of Nano-Grinding Process

**Figure 7:**
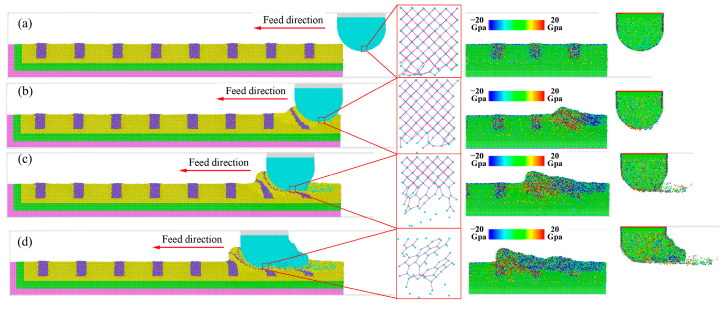
Dynamic evolution of dry grinding under different grinding distance: (**a**) 0 nm, (**b**) 8.5 nm, (**c**) 14.5 nm, (**d**) 20.5 nm.

The nano-grinding process of dry grinding was shown in Figure 7. Before grinding, the atom of workpiece came into uniform and equilibrium state after the relaxation processes, which is shown in Figure 7a. Moreover, the cementite phase showed larger internal stress, indicating that it was an unstable phase. After coming into contact with each other, the workpiece experienced severe plastic deformation, and the diamond grain showed slightly elastic deformation. It was fairly well established that the abrasive grain, on average, presented a large negative rake surface to the workpiece, and there was considerable plowing and rubbing between the abrasive grain and the workpiece along with shearing [30]. As shown in Figure 7b,c, some of the workpiece atoms were extruded to the front and side region of the abrasive grain to form ridges, while more atoms are compressed into the workpiece to form grooves. The material removal only shows the plowing, and shearing did not appear. The main reason for the absence of shearing was that the cutting depth (1.5 nm) was much smaller than the diameter of the diamond grains (10 nm), and the effective negative rake angle was larger. With the grinding distance increased, atomic strain energy rapidly accumulated, and thermal vibrations of the extra-nuclear electrons were activated, generating a high temperature. Under high stress and temperature, the carbon atoms dangling bonds at contact surface were elongated, causing the deformation of diamond lattice, and then the atomic bonds were broken, resulting in the destruction of the complete diamond lattice. Finally, the d-orbitals of iron and the p-orbitals of carbon overlapped with each other at the contact zone, forming new Fe-C bonds. Therefore, the carbon atoms can escape from their initial positions to the surface of the workpiece, leading to diffusion wear, as shown in Figure 7b.

**Figure 8:**
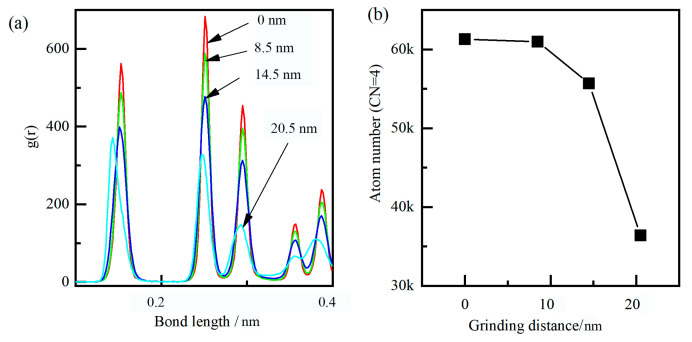
Dynamic evolution of the bond length (**a**) and the atom number (**b**) under dry grinding.

At the contact zone, carbon atoms came into long-term contact with ridges and machining surfaces, and the release of strain energy caused higher temperature on the cutting surface, resulting in the weakened bonding of diamond atoms and enhanced thermal motion of diamond atoms. Under higher stress and temperature, the covalent bond of carbon was changed from SP3 hybridization to SP2 hybridization, due to the catalysis of iron. As shown in Figure 8, the atomic bond lengths also changed from 0.157 nm to 0.146 nm, and its bond angles changed from the initial structure of 109° to 121°, which indicated that the carbon atoms transformed from a diamond lattice structure to a hexagonal structure. Moreover, as shown in Figure 7d, the strength and sharpness of the diamond grain were reduced when initial wear occurs, and the chemical wear rate was faster in the later stages.

**Figure 9:**
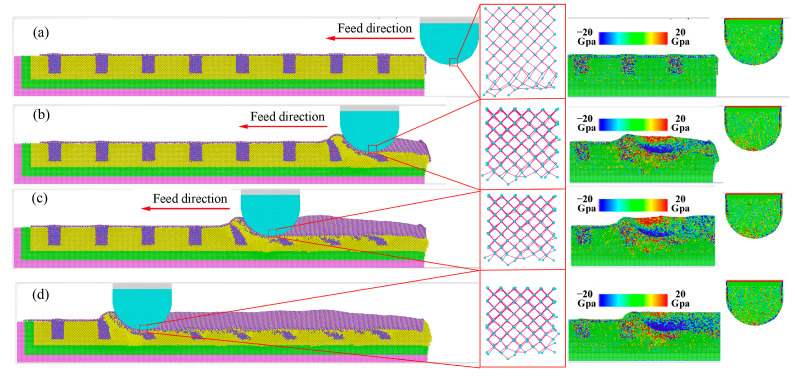
Dynamic grinding evolution under a single layer of hBN: (**a**) 0 nm, (**b**) 19.5 nm, (**c**) 36.2 nm, (**d**) 58.6 nm.

The grinding process under a single layer of hBN is shown in Figure 9. Before grinding, the single layer of hBN showed good flexibility and strength [38]. It can be adsorbed on the surface of workpiece through Pauli repulsion and van der Waals forces after the relaxation processes [39], which are shown in Figure 9a. After coming into contact with each other (Figure 9c,d), the workpiece experienced severe plastic deformation, and the diamond grain showed slightly elastic deformation, which was similar to that of dry grinding. It was fairly well established that the material removal of grinding manifested in two forms: plowing and shearing [29]. As shown in Figure 9b,c, some of the workpiece atoms were extruded to the front and side region of the abrasive grain to form ridges, while more atoms were compressed into the workpiece to form grooves. The material removal only shows the plowing, and the shearing did not appear. The main reason for the absence of shearing was that the cutting depth is much smaller than the diameter of diamond grain, and the effective negative rake angle was larger.

**Figure 10:**
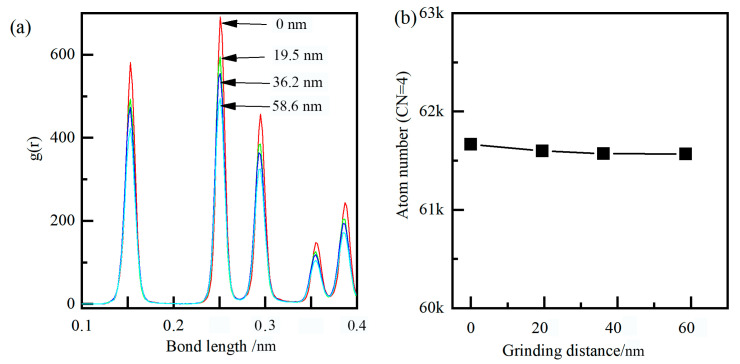
Dynamic evolution of the bond length (**a**) and the atom number (**b**) under a single layer of hBN.

Meanwhile, the flake of hBN increased the distance between the carbon and iron atom from about 0.3 nm to about 0.6 nm, resulting in the reduction of the interatomic force between the diamond grain and workpiece. Moreover, the single layer of hBN wrapped around the cutting surface forms a protective effect, which was similar to the build-up edge, and the extreme impact and alternating loads can be absorbed under this protective mechanism. Under the physical barrier, the carbon atom around the cutting surface was difficult to traverse the hBN, and almost unable to form new Fe-C bonds. As shown in Figure 10, the atomic bond length and coordinate number of diamond grain have undergone slight changes, mainly due to its plastic deformation. Therefore, as shown in Figure 9d, the diffusion and chemical wears of diamond grain can be suppressed by the hBN.

**Figure 11:**
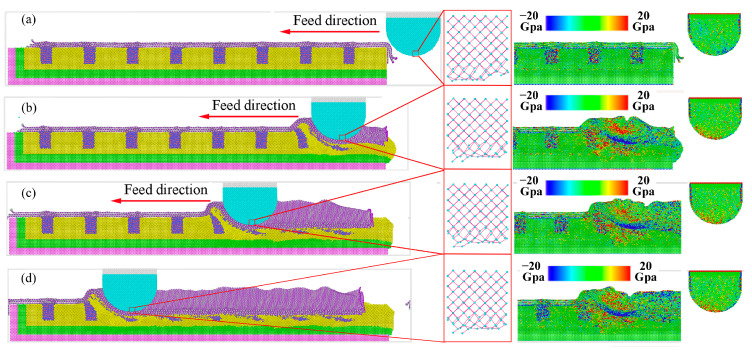
Dynamic grinding evolution under 3-layers of hBN: (**a**) 0 nm, (**b**) 19.5 nm, (**c**) 36.2 nm, (**d**) 58.6 nm.

**Figure 12:**
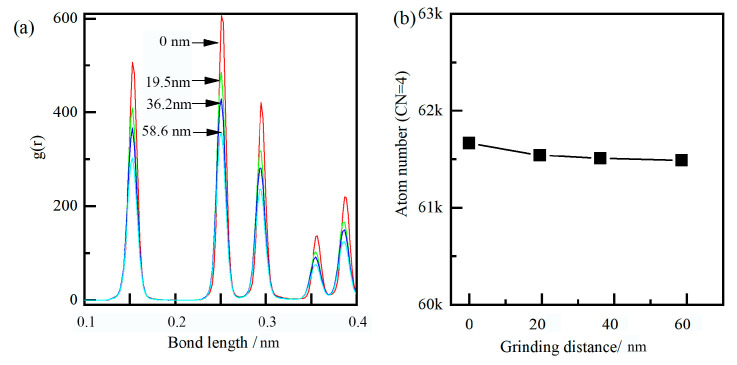
Dynamic evolution of bond length (**a**) and the atom number (**b**) under 3-layers of hBN.

Similarly to the single flake of hBN, the grinding process under the multi-layers of hBN is shown in Figure 11. As shown in Figure 11b–d, the multi-layers of hBN showed good flexibility and strength and can be wrapped around diamond grain. The multi-layers of hBN maintained the hexagonal lattice under high tensile stress (>20 Gpa), and did not experience the breakage of the B-N bond. The multi-layers of hBN can further increase the distance between a carbon and iron atom and reduce the interatomic force between the diamond grain and workpiece. At low kinetic energy, it was difficult for carbon atoms around the cutting zone to pass through hBN, resulting in lower diffusion wear. On the other hand, the stress of carbon under multi-layers of hBN was larger than that under a single layer of hBN, mainly due to the increase in effective grinding depth. As shown in Figure 12, there was almost no change in atomic bond length and coordinate number. Based on the above analysis, the hBN can effectively suppress the wear of diamond grain in the grinding process of cast steel.

### 3.3. Analysis Wear of Diamond Grain

**Figure 13:**
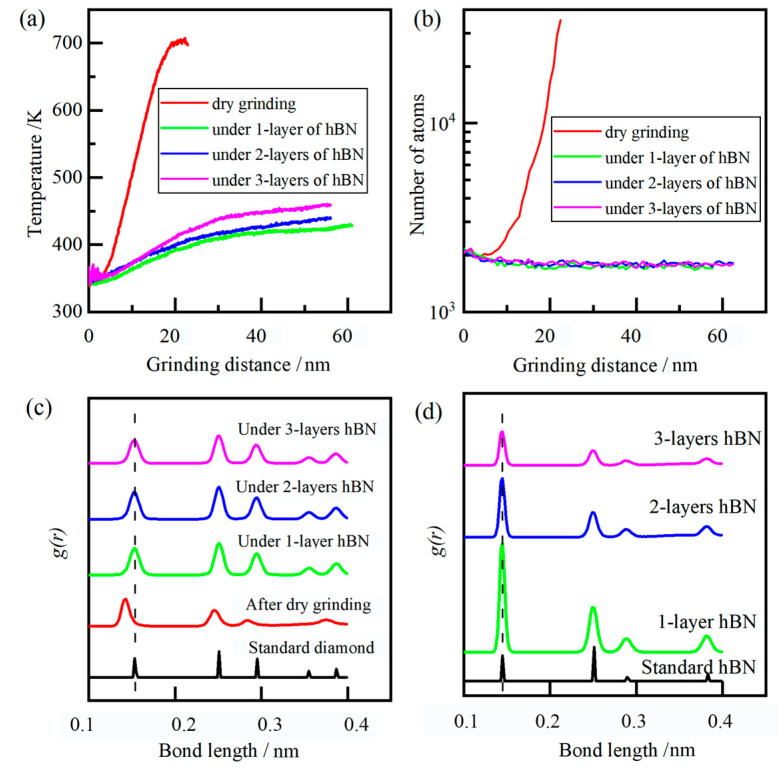
The diamond grain: temperature (**a**), the number of worn atoms (**b**), and the bond length of diamond grain (**c**), and hBN (**d**).

In the thermodynamics field, temperature was a measure of the average kinetic energy of the atom inside the diamond grain and was also an important indicator for evaluating the processing quality. As shown in Figure 13a, during dry grinding, with the increase in grinding distance, the temperature of the diamond grain rapidly grew from 300 K to 700 K. The increase in grinding temperature mainly came from the plastic deformation in the contact zone, especially the formation of hexagonal lattice, which lead to a large amount of heat generation. Higher kinetic energy could cause the C-C bond break and lead to the diffusion wear of diamond grain. When the temperature reached 700 K, the atom of diamond grain generated intense thermal motion, and its covalent bond was changed from sigma-bond to pi-bond, resulting the chemical wear of diamond grain. Under the lubrication of hBN, the temperature of diamond grain grew slowly from about 300 K to 450 K with the increase in grinding distance. This was mainly due to the flake of hBN having weakened the interatomic forces between the diamond grain and workpiece and slowed down the phonon transfer from workpiece to diamond. The temperature of diamond grain slowly grew with the increase in the number of layers of hBN.

During the grinding process, not all worn atoms of the diamond were transformed into SP2 layered six-atom ring structures. Notably, the worn atoms referred to the atoms that transformed to hexagonal lattice in diamond grain, or the atoms that detached from diamond grain and formed Fe-C bonds. In order to evaluate the grain wear, the coordination number of diamond grain (Figure 13b, coordination number < 4) was monitored and analyzed. In the initial elastic contact stage, the number of worn atoms showed almost no change at different grinding conditions, indicating that it was a stable phase at low temperature and pressure. At stable grinding stage, the diamond grain underwent diffusion and chemical wear in sequence under dry grinding conditions, and the number of worn atoms grew exponentially with the grinding distance, indicating that it was prone to phase transition, especially under high temperatures and iron catalysis. On the other hand, during lucubration of hBN, the number of worn atoms showed slight fluctuations and also showed almost no change. The slight fluctuations of the worn atoms were mainly from the grain surface due to the random phonon–phonon collision.

The radial distribution function (RDF) was a primary linkage between macroscopic thermodynamic properties and intermolecular interactions. After grinding, the RDF of diamond grain and hBN are shown in Figure 13c,d. The peaks of standard hBN (at 0.1449, 0.2511, and 0.2899 nm) corresponded to the nearest neighbor distance of B-N, B-B/N-N, B-N, respectively. After grinding, the peak position had almost no change, and the peak width was broadened, indicating that the hBN was a stable phase and no phase transformation under large plastic deformation occured. The main peak positions of standard diamond were 0.154, 0.251, and 0.295 nm, corresponding to the near neighbor distance. After dry grinding, the peak position shifted from 0.154 nm to the left 0.142 nm, indicating that the diamond content had decreased to a certain extent, while the newly formed graphite content had increased. After lubrication of hBN, the peak position showed almost no change, and the peak width was broadened, indicating no phase transformation. Based on the above analysis, the diffusion and chemical wear of diamond grain can be suppressed under the lubrication of hBN.

According to the above simulation analysis, under dry grinding, the diamond grain experienced serious diffusion wear, and carbonization, in turn, which was consistent with the experiment observation in Figure 4 where the diamond grain showed serious the abrasive wear. During the lubrication grinding, the flake of hBN maintained a hexagonal lattice under plastic deformation, with slight changes in bond length, indicating that the hBN was a stable phase. Under the lubrication of hBN, the flake of hBN could increase the distance between carbon and iron atoms, leading to a decrease in interatomic force between diamond grain and the workpiece. Moreover, the flake of hBN can prevent carbon atoms from passing through and reaching the surface of the workpiece, thus preventing the formation of new Fe-C chemical bonds. So, the wear of diamond grain, such as diffusion wear, and carbonization were suppressed. Under water-based hBN spray, the diamond grain (Figure 6) showed slightly broken wear, which was due to the periodical alternating mechanical and thermal stress, and there was almost no abrasive wear, which was consistent with the simulation analysis.

### 3.4. Analysis Stress of Diamond Grain

**Figure 14:**
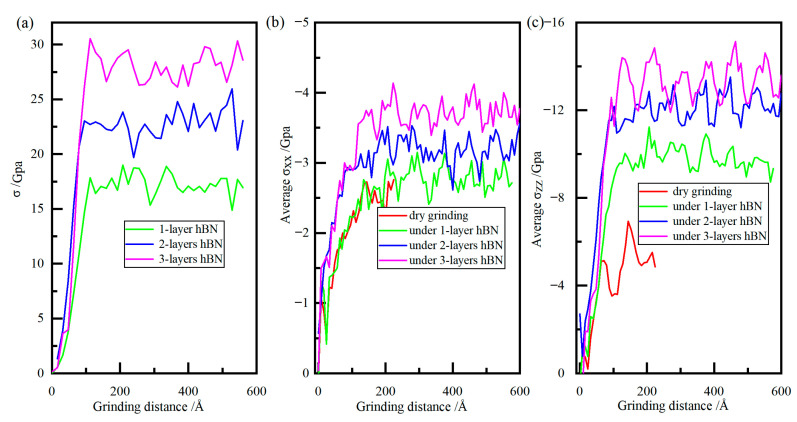
The hydrostatic stress of hBN (**a**), the stress of diamond grain along *x*-axis (**b**), and the stress of diamond grain along *z*-axis (**c**).

During the grinding process, the hBN around the contact zone underwent serious deformation, and von Mises stress was applied to evaluate its deformation behavior, which was shown in Figure 14a. With the advancement of abrasive grain, the value of stress first increased rapidly and then tended to be stable. The stress of hBN increased with the increase in hBN layer, due to the increase in effective grinding depth. During the grinding process, the hBN maintained the hexagonal lattice due to the stress being lower than its tensile strength (100 GPa), which agreed with Figure 11d. The average stress components of diamond grain were shown in Figure 14b,c, where σ_xx_ and σ_zz_ were the normal stresses in the x and z directions. Evident from Figure 14, the σ_xx_ and σ_zz_ was lower than zero, indicating a compressive stress, which was consistent with the situation of extrusion removal. With the advancement of abrasive grain, the value of σ_xx_ and σ_zz_ first increased rapidly and then tended to be stable. At the stable stage, the value of σ_xx_ and σ_zz_ showed a certain degree of fluctuation, mainly due to the composition of the hard phase of cementite and the soft phase of ferrite in the workpiece. Meanwhile, the value of σ_xx_ and σ_zz_ increased with the increased number of hBN layers, mainly because that the volume of removed material increased with the increase in effective grinding depth.

**Figure 15:**
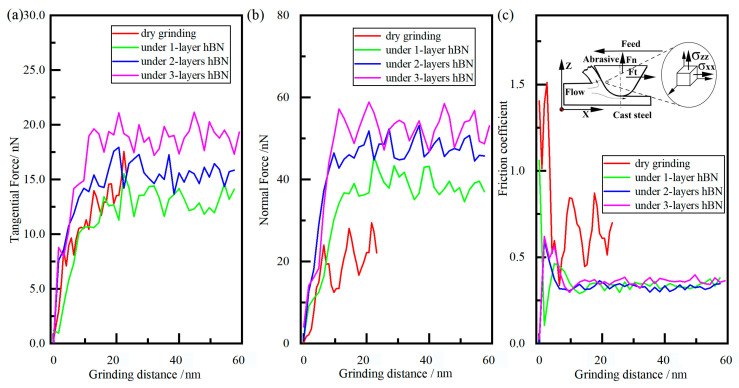
The tangential force (**a**), normal force (**b**), and friction coefficient (**c**) of diamond grain under different conditions.

During the grinding process, the grinding force was generated by elastic deformation and plastic deformation, which is shown in Figure 15. Similarly to the stress trend, the tangential and normal forces first increased rapidly and then tended to be stable. The normal forces were much larger than the tangential force due to the large negative front angle of diamond grain, which was consist with previous reports [12]. As shown in Figure 15c, when the grinding condition changed from dry to lubricated, the value of the friction coefficient was decreased from approximately 0.5 to 0.25, indicating that the flake of hBN possessed good lubrication performance. From the above simulation analysis, the flake of hBN layer can not only suppress the chemical wear of diamond grain, but also decrease the friction force, which can be used to the grinding field.

## 4. Conclusions

In order to suppress the wear of diamond grain, this paper proposed the novel water-based hBN as a minimum quantity lubrication in the grinding process of cast steel and conducted the grinding experiment and molecular dynamics simulations under different conditions. Based on the experimental and simulation results, we drew the following conclusions relevant to the wear suppress behavior:

(1) The experiment results show that compared to dry grinding, the water-based hBN nanofluid can effectively reduce the maximum temperature of the workpiece at the contact zone from 408 K to 335 K and cause diamond grains to change from severe abrasion wear to slightly micro-broken wear.
(2) The molecular dynamics indicate that the flake of hBN can weaken the catalytic effect of cast steel on the diamond, preventing the diffusion of carbon atom to cast steel and suppressing the graphitization of diamond grain.
(3) Under higher tensile stress, the flake of hBN can maintain the hexagonal lattice, improve the contact state between the diamond grain and cast steel and reduce the cutting heat and friction coefficient from about 0.5 to 0.25.

The results indicated that the proposed water-based hBN as the MQL was an effective way to reduce the wear of diamond grain in grinding of cast parts. Moreover, the water-based hBN as the MQL also promoted environmentally conscious manufacturing. In future research work, some useful exploration attempts shall be made on the grinding application of diamond wheel on black metal fields such as the structures of large engineering, construction steel, and rail transit based on the current experimental and theoretical findings.

## Figures and Tables

**Table 1:** Grinding simulation parameters.

Parameters	Value
Workpiece material	Cast steel: 88% ferrite (BCC lattice, 0.286 nm); 12% cementite (orthorhombic lattice, 0.50 × 0.45 × 0.67 nm)
Size of simulation area	90 × 20 × 14 nm
Abrasive grain	Diamond, cubic lattice, 0.3567 nm
Size of abrasive grain	Sphere (diameter 10 nm), cylinder (diameter 10 nm, height 3 nm)
Boron nitride	Hexagonal lattice, 0.25 nm; Size: 70 nm × 17 nm
Time step	0.001 ps
Equilibration temperature	300 K
Relaxation time	120 ps
Grinding direction	(−1 0 0)
Grinding speed	30 m/s
Depth of cut	1.5 nm

## Data Availability

The data presented in this study are available upon request from the corresponding authors.
